# Reduction of Vascular Inflammation, LDL-C, or Both for the Protection from Cardiovascular Events?

**DOI:** 10.2174/1874192401812010029

**Published:** 2018-03-30

**Authors:** Andromachi Reklou, Michael Doumas, Konstantinos Imprialos, Konstantinos Stavropoulos, Dimitris Patoulias, Vasilios G. Athyros

**Affiliations:** 1 Second Propedeutic Department of Internal Medicine, Medical School, Aristotle University of Thessaloniki, Hippocration Hospital, Thessaloniki, Greece; 2 George Washington University, Washington, DC, USA

**Keywords:** LDL-C, Inflammation, Cardiovascular events, Statins, Ezetimibe, PCSK9 inhibitors

## Abstract

**Background:**

Low density lipoprotein cholesterol (LDL-C) and low grade arterial inflammation are key pathogenic factors for atherosclerosis and its manifestation, cardiovascular disease (CVD).

**Objective:**

In this narrative review we assessed if decreasing LDL-C levels or inflammation or both is more effective in reducing CVD events.

**Results:**

In the Scandinavian Simvastatin Survival Study (4S), all statin trials of the 90s’ and the Further Cardiovascular Outcomes Research with PCSK9 Inhibition in Subjects with Elevated Risk (FOURIER) the benefit came from the LDL-C reduction. In the GREak and Atorvastatin Coronary heart disease Evaluation (GREACE), the Treating to New Targets (TNT), and the Justification for the Use of Statins in Prevention: an Intervention Trial Evaluating Rosuvastatin (JUPITER) trials both mechanisms in combination produced significant benefits. In the Atorvastatin for Reduction of MYocardial Damage during Angioplasty (ARMYDA) trials and the Canakinumab Antiinflammatory Thrombosis Outcome Study (CANTOS) with a human antibody targeting IL-1β with no lipid lowering effect, the reduction in arterial inflammation played the only beneficial role because there was no change in lipids levels.

**Conclusion:**

Both LDL-C and inflammation reduction are beneficial to the reduction of CVD risk. However, canakinumab is a very expensive drug that only induced a 15% reduction in CVD events, thus drastically reducing the possibility for it to be used in clinical practice. Besides, canakinumab is associated with increased infections, some fatal. A potent statin with anti-inflammatory effects is probably the best choice for the majority of those needing hypolipidaemic drug therapy.

## 1 INTRODUCTION

The year 1994 was a defining one for cardiovascular risk disease (CVD) prevention. The prospective, randomised, double blind, placebo controlled, Scandinavian Simvastatin Survival Study (4S) showed in 4,444 patients with coronary heart disease (CHD) that simvastatin treatment (mean dose 26 mg/d) reduced total mortality by 30%, CVD events by 34%, and the need for revascularization by 37% (all significant) [1]. This substantially changed clinical practice in regard to secondary CVD prevention [1]. The main function of simvastatin was the safe reduction in low density lipoprotein cholesterol (LDL-C) levels by 35%, while high density lipoprotein cholesterol (HDL-C) levels increased by 8% [1]. Therefore, the clinical benefits of 4S were attributed mainly to the magnitude of LDL-C reduction by simvastatin, thus contributing to future guidelines [1, 2]. Data from the same study supported that a 1% reduction in LDL-C reduces major coronary events (MACE) risk by 1.7% (95% CI, 1.0-2.4%; p<0.00001) [2]. These findings were verified by other epidemiological studies [3-5] and the guidelines of the time [6, 7] emphasized the reduction of LDL-C levels and set treatment targets using LDL-C levels according to CVD risk [3-7].

Some researchers could not attribute the reduction of total mortality only to LDL-C statin-induced reduction, and tried to explain this benefit with “off target” effects of statins. For example, on inflammation, chlamydia pneumoniae, helicobacter pylori or anti-oxidized LDL antibodies were proposed as risk factors and possible targets of hypolipidaemic therapy [8]. In a 2002 *post hoc* study, simvastatin reduced the increased baseline high sensitivity C reactive protein (hsCRP; a marker of inflammation) levels, but it did not affect the other 3 suggested total mortality risk factors (seropositivity for Chlamydia pneumoniae or Helicobacter pylori, nor the levels of anti-oxLDL antibodies) in patients with stable CVD at baseline [8]. Simvastatin treatment reduced hsCRP levels, but did not have an effect on increased baseline CVD risk [8]. Despite the fact that the reduction of hsCRP by simvastatin had no significant clinical impact, this was enough to open the discussion about the possible beneficial clinical effects of statins by reducing coronary artery inflammation, which is implicated in the pathogenesis of atherosclerosis and contributes to the manifestation of clinical events [9-12].

## 2 LDL-C REDUCTION AND THE DECREASE IN CVD RISK

After the 4S study, other interventional studies followed using several statins (*e.g.* simvastatin, pravastatin, atorvastatin, rosuvastatin) [13-20]. The conclusion of these studies was that statins safely reduce CVD morbidity and mortality in different types of high-risk patients [men, women, hypertensives, patients with diabetes mellitus (DM) with or without metabolic syndrome (MetS), subjects with or without baseline overt CVD], irrespective of initial LDL-C levels [13-20]. The benefit was expressed as a substantial reduction in the rates of myocardial infarction (MI), stroke or coronary revascularization [13-20]. Therefore, among the different high CVD risk situations, statin administration would protect about 70-100/1000 patients on the drug from a major CVD event, while longer treatment should produce further benefit [15-17]. The size of the 5-year benefit depended mainly on individual overall risk of major CVD, rather than on their blood lipid concentrations alone [15]. The variance in LDL-C reduction following statin therapy is wide and the % reduction directly relates to CVD outcomes [19]. The results of all these studies, placebo or active treatment group controlled, showed that there is a linear relation between LDL-C levels and CVD events, suggesting a strong relationship.

These data support guideline approaches that incorporate both % reductions of LDL-C targets for statin therapy as well as absolute LDL-C targets, providing room for PCSK9 inhibitor use to further reduce CVD events [19].

There were 4 meta-analyses published during a period of 7 years (2005-2012) that analysed the efficacy and safety of cholesterol-lowering treatments from data coming from 90,056 participants in 14 randomised statin trials [21], efficacy and safety of more intensive lowering of LDL-C with data from 170,000 participants in 26 randomised trials [22], while the third meta-analysis evaluated the effects of LDL-C reduction with statin therapy in people at low risk of CVD with data coming from 134,537 subjects from 27 randomised trials [23]. All meta-analyses concluded that a LDL-C reduction by 1 mmol/l (39 mg/dl) resulted in a CVD event reduction between active treatment and control, mainly placebo, from 21-24%, with the reduction in CVD events being greater (paradoxically) in low risk patients than the high risk ones [21-23]. If the reduction was higher than 1 mmol/l the CVD event decrease might be as high as 40-50% [22]. The other conclusion was that CVD risk reduction only correlated with LDL-C reduction [21-23].

Finally, a meta-regression analysis of 312,175 participants from 49 trials with 39,645 major CVD events [24] showed that statin use and non-statin therapies that reduce LDL-C to the same degree were associated with similar relative risk reductions in CVD events per mmol/l change in LDL-C, both in primary and in secondary CVD prevention, and lower achieved LDL-C levels were associated with lower rates of major CVD events for both statin or non-statin therapies [24].

In the Improved Reduction of Outcomes: Vytorin Efficacy International Trial (IMPROVE-IT), involving 18,144 patients with acute coronary syndrome (ACS), the addition of ezetimibe to statin therapy, resulted in incremental lowering of LDL-C levels and improved CVD outcomes [25]. Moreover, lowering LDL-C to levels below previous targets provided additional clinical benefits [25]. This was the first time LDL-C reduction by a non-statin drug resulted in CVD event reduction [25]. This reinforces the concept that LDL-C reduction translates to CVD benefit regardless of the way of its reduction [25]. This was also shown in the Further Cardiovascular Outcomes Research with PCSK9 Inhibition in Subjects with Elevated Risk (FOURIER) [26] trial (n= 27,564 high risk patients), where inhibition of PCSK9 with evolocumab (140 mg every 2 weeks or 420 mg monthly) on top of statin (and ezetimibe in some patients) therapy lowered LDL-C levels to a median of 30 mg/dl (0.78 mmol/l) and substantially reduced the risk for CVD events [26]. These findings show that patients with atherosclerotic CVD benefit from the LDL-C lowering, below current targets, regardless of the intensity of statin therapy or ezetimibe use [26].

The above suggest in the 2016 European Guidelines that LDL-C reduction plays a vital role in CVD risk reduction in all groups of patients, regardless the drug used (statin, ezetimibe, PCSK9 inhibitors or combinations) [27]. This is probably the reason that most (if not all) guidelines set LDL-C targets, according to the baseline CVD risk level of patients [27]. However, some researchers suggest that there are pleiotropic effects of statins (anti-inflammatory properties, improvement of endothelial dysfunction, increased nitric oxide bioavailability, antioxidant effects and plaque stabilization) that contribute to the earlier or greater reduction of atherosclerotic risk as expressed by the incidence of CVD events [28]. Data from the Cholesterol and Recurrent Events (CARE) trial suggest that “Evidence of inflammation after MI is associated with increased risk of recurrent coronary events and therapy with pravastatin may decrease this risk, an observation consistent with a non-lipid effect of this agent.” [29].

## 3 INFLAMMATION REDUCTION FOR THE DECREASE OF CVD RISK

Elevated levels of markers for inflammation such as hsCRP, interleukin-6, and intercellular adhesion molecule-1 (ICAM-1), have been related with increased risk for both first and recurrent CVD events [29, 30]. hsCRP levels, in particular, appear to be the most potent predictor of future CVD events [29, 30].

## 3.1 GREACE Atorvastatin and Coronary Heart Disease Evaluation (GREACE) Study

In GREACE we enrolled 800 patients with CHD in the usual care group and 800 patients with CHD in the structured care group, who were administered atorvastatin 10-80 mg once daily [forced titration of atorvastatin to reach the LDL-C target of <100 mg/dl (2.6 mmol/l), the treatment target of the period] [16]. The mean daily dose was 24 mg [16]. Atorvastatin induced a 46% mean reduction in LDL-C levels, 44% in non-HDL-C levels and 31% in triglycerides (TGs), while the mean increase in HDL-C levels was 7% (p<0.001 vs. baseline, for all parameters). During the 3-year duration of the study, 12% of all patients on atorvastatin had a CHD recurrent event, compared with 24.5% of all patients in the usual care group (relative risk reduction, RRR, 51%, p<0.0001). Moreover, atorvastatin led to a 43% decrease in total mortality (p=0.002), a 47% reduction in CHD mortality (p=0.002), a 54% decrease in CHD morbidity, myocardial infarction (MI), and coronary revascularization (p<0.0001) and in a 47% reduction stroke incidence (p=0.034) [16, 31].

A *post hoc* analysis of GREACE examined the effect of statins *vs*. untreated dyslipidaemia on renal function in patients with CHD [32]. Atorvastatin administration increased estimated glomerular filtration rate (eGFR) by 12% until the end of the 3 year period of the study [32]. At the time atorvastatin was the only drug administered for a chronic disease that increased eGFR [32]. However, this increase became statistically significant by the 6th week of treatment with atorvastatin [32]. Regression of possible atheromatic plaques in renal arteries, arcuate arteries, or afferent arterioles is not likely to be achieved as early as the 6th week of treatment and commonly takes years to occur [32]. The eGFR improvement was attributed to the decrease of renal arterial wall inflammation, to the reduction of glomerulosclerosis (a procedure analogous to atheromatic plaques that might develop early as shown in patients with diabetic nephropathy or in animal models), because mesangial cells have binding sites for LDL particles that might cause inflammation of the glomerulus, as well as to the increased nitric oxide bioavailability leading to endothelial-related vasodilatation [32-34]. This increase in eGFR by atorvastatin had a clinical impact since with every 5% increase of eGFR, a 16% (p=0.003) reduction in CVD events was recorded while with every 5% decrease (seen in the usual care patients not on statins) [32] there was a 10% increase in CVD events, after adjustment for several CVD risk factors, among which LDL-C reduction [32]. This is probably the first case where the effect of a statin on inflammation induced earlier and greater clinical benefits [32]. Thus, eGFR reduction caused an early and substantial improvement in CVD outcomes, irrespective of LDL-C reduction, probably due to the pleiotropic effects of statins (among which is the reduction in inflammation) [32]. At the 6^th^ week when eGFR was significantly increased, all patients on atorvastatin were on a 10 mg/d dose [16]. After that, a dose titration was performed until the 6^th^ month to the point that the LDL-C target (<100 mg/dl, 2.6 mmol/l) was attained by nearly all patients. During this period (with a higher dose of atorvastatin) there was a greater increase in eGFR (i.e. the anti-inflammatory effect might be dose dependent). Of note, when patients achieved the LDL-C target, a further increase in eGFR was noted [32]. This could be attributed to LDL-C reduction (that needs time to be expressed clinically) and this is probably the first case where reduction of clinical outcome was first achieved by the anti-inflammatory effect of the statin followed by a hypolipidaemic one: a combination of pleiotropic and hypolipidaemic effects of statins [32].

These results were confirmed by the Treating to New Targets (TNT, n=10,001 CHD patients followed for 5 years) programmed *post hoc* analyses [35-37]. TNT compared atorvastatin 80 vs 10 mg/d [20]. The anticipated 5-year decline in eGFR was not observed; on the contrary both an absolute CVD event reduction/1 ml/min/1.73 m^2^ increase in eGFR of 2.0% was reported with 10 mg/d of atorvastatin and 3.3% with 80 mg/d of atorvastatin was noted, suggesting this effect may be dose-related [35]. The same occurred in patients with chronic kidney disease (CKD) (eGFR was increased up to 10% by the 80 mg/d dose) [37]. The study found that CHD patients have progressive CKD and that they are at high risk for future CVD events [36]. The early absolute and relative increases in eGFR with atorvastatin in high risk patients was associated with a reduction in CVD events much greater than in any other previous secondary CVD prevention study [35, 37], similar to the one recorded in the *post hoc* analysis of GREACE [32].

Similar were the effects of atorvastatin in patients with CHD and metabolic syndrome [38]. All the above suggest that first the reduction in inflammation and afterwards the reduction in LDL-C have a beneficial effect that improvers renal function, a potent CVD risk factor, and reduces CVD events, independently of the initial lipid profile improvement.

### 3.2 The Justification For the Use of Statins in Prevention: An Intervention Trial Evaluating Rosuvastatin (JUPITER) Trial Conclusions

In the JUPITER trial 17,802 healthy men and women with LDL-C levels of <130 mg/dl (3.6 mmol/l) and hsCRP levels of ≥2.0 mg/l or higher were randomized to 20 mg/d rosuvastatin or placebo [39] During the study rosuvastatin reduced LDL-C levels by 50% and hsCRP levels by 37% [39]. At the end of the study (1.9 years) rosuvastatin showed a reduction in the composite CVD endpoint by 47% (p<0.0001) [39]. This means that in healthy persons without hyperlipidaemia but with elevated hsCRP, rosuvastatin significantly reduced the incidence of major CVD events [39]. In TNT, a benefit was reported even in women with a relative risk reduction similar to that in men [40], and in older persons with the same characteristics as the participants of the main study [41]. The study also showed that increased levels of hsCRP without statin therapy remaining high over time are related to CVD manifestation and progression [42]. A paper based on JUPITER provided data supporting that increased levels of GlycA (a novel protein glycan biomarker of N-acetyl side chains of acute-phase proteins) were associated with an increased risk of CVD events independent of traditional risk factors and hsCRP [43]. The Cardiovascular Inflammation Reduction Trial (CIRT) is a randomized clinical trial investigating whether taking low-dose methotrexate reduces CVD in people with T2DM or MetS that have had an MI or multiple coronary blockages. This trial is funded by the National Heart, Lung, and Blood Institute (NHLBI)/National Institutes of Health (NIH) https://clinicaltrials.gov/ct2/show/NCT01594333. This trial will verify the inflammation theory suggested by JUPITER.

In any case, the aforementioned data suggest that the concomitant reduction of LDL-C and vascular inflammation (as mirrored by a significant hsCRP reduction) [39-44] had a substantial benefit on CVD events, greater than any primary prevention study before with any statin. The clinical benefit was significantly greater when both LDL-C and CRP levels were reduced than when only one of the variables was lowered to the same level [23, 25, 29, 39].

### 3.3 The Acute Coronary Syndromes (ACS) Trials

In the initial large statin survival trials patients with ACS were not included, because they had a higher mortality rate as compared with patients with chronic stable CHD and this might lead to confused results [44]. In 2001 the Myocardial Ischemia Reduction with Aggressive Cholesterol Lowering (MIRACL) Study was reported [44]. In this study, 3,086 patients with unstable angina or non-Q-wave acute MI were randomised either to 80 mg/d of atorvastatin or placebo [44]. LD-C was reduced from 124 mg/dL (3.2 mmol/L) at baseline to 72 mg/dL (1.9 mmol/L) 4 months later; hsCRP was reduced by 34% (p<0.001) [44]. During the study (4 months) a 16% (p=0.048) reduction in recurrent CVD events by atorvastatin compared with placebo during the study, was observed [44]. In MIRACL an increase in tPA concentration was related to a higher early risk of recurrent CVD events [45], however, it is unlikely that statin benefit was achieved through thrombolytic and fibrinolytic pathways, suggesting a greater role in anti-inflammatory effect [45].

Three years later the first study comparing two different statins, head to head, at their higher doses in patients with ACS was reported: the Pravastatin or Atorvastatin Evaluation and Infection Therapy-Thrombolysis in Myocardial Infarction 22 (PROVE IT) [46]. The study included 4,162 patients who had an ACS within the last 10 days and compared 40 mg of pravastatin daily (standard therapy) with 80 mg of atorvastatin daily (intensive therapy) [46]. In study participants with ACS, atorvastatin therapy provided greater protection against death or major CVD events than pravastatin; a 16% reduction in the hazard ratio (HR) in favour of atorvastatin was found [p=0.005; 95% confidence interval (CI), 5-26%) [46]. During the first month the RRR between the two treatments was 33% (p=0.043) [46]. In the pravastatin group the achieved LDL-C was 95 mg/dL (2.5 mmol/l) and in the atorvastatin group the achieved LDL-C of 62 mg/dL (1.6 mmol/l), a 30% difference (p<0.001), while atorvastatin reduced hsCRP levels 38% more than pravastatin (p<0.001) [46]. A Cochrane analysis suggests that this benefit of statin treatment is compound specific and not a drug class effect [47]. In a *post hoc* analysis of PROVE IT, it was shown that those patients (from the atorvastatin group mainly) who achieved the double goal of LDL-C <70 mg/dl (2.6 mmol/l) and hsCRP <1 mg/l had a 60% reduction (p<0.0001) in CVD events as compared with those that did not achieve either goal [48]. These results indicate that ACS patients benefit from potent statins to achieve LDL-C levels (and hsCRP reduction) considerably lower than those suggested by the guidelines targets at that time [48].

However, there was a failure to achieve better clinical results in ACS patients with the high dose of simvastatin *vs.* a lower dose of the same statin [49]. The Agrastat and Zocor (A to Z) trial was a randomized, double-blind trial of patients that had an ACS [49]. Participants were randomized to 40 mg/d of simvastatin for 1 month and then 80 mg/d afterwards (n=2,265) and were compared with ACS patients receiving placebo for 4 months and 20 mg/d of simvastatin afterwards (n=2,232) [49]. A total of 16.7% in the placebo-20 mg/d of simvastatin group and 14.4% in the 40 mg/80 mg simvastatin group had a primary end point (HR, 0.89; 95% CI 0.76-1.04; p=0.14) [49]. CVD death occurred in 5.4% and in 4.1% of the patients in the 2 groups, respectively (HR, 0.75; 95% CI, 0.57-1.00; p=0.05) but no differences were observed in other individual components of the primary end point [49]. Throughout the first 4 months there were no differences between the groups for the primary end point (HR, 1.01; 95% CI, 0.83-1.25; p=0.89), but from the 4^th^ month of the study till the end of the study (20 months) the primary end point was significantly reduced in the high dose simvastatin group (HR, 0.75; 95% CI, 0.60-0.95; p=0.02) [49]. The trial did not achieve the prespecified end point overall [49]. The explanation that tried to clarify these findings comes from an editorial by a different research group [50]. The first 4 months need a substantial reduction in inflammation, while afterwards (during the next 20 months) the LDL-C reduction is helpful [50]. There was a significant difference between the hsCRP between the 3 ACS studies: In MIRACLE the difference between groups was 34%, in IMPROVE IT 38% and in A to Z it was half or less (17%) [50]. Additionally, PCSK9 inhibitors (PCSK9i), novel and powerful lipid-lowering agents, have been used in this clinical setting [51 https://clinicaltrials.gov/ct2/show/NCT01663402]. In this review, we summarize the present statin therapy, and refer to ezetimibe and PCSK9i as novel or additional non-statin strategies in the management of ACS [51].

In any case the data above mean that the concomitant reduction of LDL-C and vascular inflammation (as represented by a significant hsCRP reduction) had a substantial benefit on CVD events, greater than any ACS study before with any statin.

### 3.4 The Atorvastatin For Reduction of MYocardial Damage During Angioplasty (ARMYDA) Trials

Considerable myocardial necrosis, as indicated by a significant increase in creatine kinase-MB (CK-MB), is present after 40% of percutaneous coronary intervention (PCI) cases [52, 53]. Although most patients remain asymptomatic with no changes in cardiac function, even a mild release of CK-MB identifies a population with a worse long-term prognosis compared with patients with no enzyme elevations (higher mortality during follow-up) [53-55]. After a longer follow-up period it was found that even a small increase in CK-MB after PCI is associated with a small, statistically and clinically significant, increase in the risk of CVD death [55]. Many treatment strategies have been proposed to address this issue, but procedural ischemic myocardial injury remains a frequent complication after coronary angioplasty [54].

CHD is an insidious public health problem that reduces life expectancy and quality of life, worldwide [56]. PCI has produced satisfactory outcomes, thus becoming a major approach for CHD therapy [57]. In antithesis to impeccable preoperative preparation of the patient with drugs and effective intra-operative procedures, postoperative myocardial injury remains a big problem for PCI [58]. During the last few years it was shown that post-procedural side effects were related to coronary inflammation and the benefit was expressed by a statin effect (24 h after the PCI, abnormal cTnI and TNF-α and IFN-γ levels in the experimental group was remarkable reduced *vs* than the control group, while IL-10 was increased) [59, 60]. That is why statins are used prior to PCI to have an early dose-dependent inflammation related benefit, not dependent on their hypolipidaemic activity [61].

Based on this idea a group from Italy performed a series of studies, the ARMYDA studies, by administrating a statin before PCI [62-69]. The first study included 143 statin naive patients with stable CHD and randomized them to atorvastatin (40 mg/d, n=76) or placebo (n=77) 7 days before the procedure [62]. After PCI the increase of markers of myocardial injury were 12 *vs* 35% for CK-MB (p=0.001), 20 *vs* 48% for troponin I (p=0.0004), and 22 *vs* 51% for myoglobin (p=0.0005) [62]. MI by CK-MB determination was detected after PCI in 5% of patients in the atorvastatin group and in 18% in the placebo group (p=0.025) [62]. All the above suggest that pretreatment with 40 mg/d of atorvastatin for 7 days significantly reduces post-procedural myocardial injury in elective PCI [62]. These results are attributed to the anti-inflammatory properties of atorvastatin [62, 63]. The same results were seen with treatment with atorvastatin 40 mg/d, initiated 7 days before coronary bypass; a significant reduction in the occurrence of postoperative atrial fibrillation (AF) (which occurs in 10-65% of patients postoperative and is related with cardiac inflammation) was also observed [64-67]. Atorvastatin use was also found to shorten the hospitalization period [64]. These results may have clinical implications in cardiac surgery [64]. In another ARMYDA Trial (the ARMYDA-ACS trial) short-term pretreatment (7 days) with atorvastatin may improve outcomes in patients with ACS undergoing early PCI; 88% reduction in post procedural MI, p=0.004 [68]. In another ARMYDA study (ARMYDA-EPC) 80 mg of atorvastatin 12 h before and 40 mg 2 h before PCI in patients on long-term statin therapy increased endothelial progenitor cells (EPCs) that may induce vascular repair, which are able of early colony formation and may contribute to cardioprotection [69]. All the above ARMYDA trials were performed in statin naive patients. However, the ARMYDA-RECAPTURE trial was performed in patients already in statins [70]. Administration of 80 mg atorvastatin 12 h before PCI, with a further 40 mg pre-procedural dose produced substantial reductions in the primary endpoint (cardiac death, MI, or revascularization) [3.7 of *vs* 9.4% in the placebo arm (p = 0.037)] [70].

All the above benefits are attributed to the anti-inflammatory effects of atorvastatin [62-70]. A systematic review and meta-analysis showed that, compared with the low dose, the high dose of rosuvastatin (20 mg/d) was more beneficial to patients with ACS in China and should be preferred vs the low dose [71]. A study with rosuvastatin loading in the elderly with an ACS that had a PCI can attenuate the increase in hsCRP, CK-MB, and cTnI, decrease myocardial injury and inflammatory reaction caused by PCI, and improve left ventricular function 30 days after PCI [72]. Similar were the effects of all potent statins [73]. Moreover, in patients that undergo PCI, statin therapy is effective at reducing the risk of contrast-induced acute kidney injury and its short- mid- long-term adverse effects related to CVD events [74-77]. Thus, this should be considered, at least on a short-term basis, for patients at increased risk of this complication [74-78].

### 3.5 Canakinumab Antiinflammatory Thrombosis Outcome Study (CANTOS) Trial

There are data from the statin trials suggesting that low-grade arterial wall inflammation plays a causal role in the pathogenesis of atherosclerosis and its CVD manifestation; however, drug trials targeting inflammation have not yet shown clinical benefit. The CANTOS trial investigated the effects of canakinumab (Ilaris^®^), a fully human monoclonal antibody that inhibits interleukin-1β, a cytokine vital to the inflammatory response [79, 80]. CANTOS included 10,061 patients with previous MI and an hsCRP ≥2 mg/l [79]. Three doses of canakinumab targeting the interleukin-1β innate immunity pathway (50, 150 and 300 mg, injected subcutaneously every 3 months) were compared with placebo on top of usual treatment [79]. The primary endpoint was nonfatal MI, nonfatal stroke, or CVD death [79]. At 4 years, the median reduction from baseline in hsCRP compared placebo was 26% in the 50 mg dose of canakinumab, 37% in the 150 mg group, and 41% in the 300 mg group, while there was no reduction in lipid levels with any dose [79]. CVD events were reduced *vs*. placebo by 7% in the 50 mg group (p=0.30), 15% in the 150 mg group (p=0.021) and 14% in the 300 mg group (p=0.031). Only the 150 mg dose met the prespecified multiplicity-adjusted statistical significance for the primary and secondary (included hospitalization for unstable angina) [79]. There was no significant difference in all-cause mortality (6% reduction, p=0.31) [79]. However, canakinumab was associated with a non-significant higher incidence of fatal infection compared with placebo (6%, p=0.31) [79]. Neutropenia was more common among patients who were on canakinumab than among those on placebo, and significantly more deaths were attributed to infection or sepsis in the pooled canakinumab groups than in the placebo group (incidence rate, 0.31 vs. 0.18/100 person/year; p=0.02) [79]. Thrombocytopenia was more common among patients on active treatment than on placebo, but no significant difference in the incidence of haemorrhage was observed [79].

The current cost of canakinumab in the U.S. may be a major problem for the generalization of such a treatment (≈$65,000 annually for the 150 mg dose) [https://www.jwatch.org/na44910/2017/08/27/ anti-inflammatory-therapy-atherosclerotic-disease-step]. The side effect of fatal infection requires additional investigation, as does the decrease by canakinumab incidence of arthritis, gout and fatal cancers [https://www.jwatch.org/na44910/2017/08/27/ anti-inflammatory-therapy-atherosclerotic-disease-step]. Further research may identify agents with superior antiatherosclerotic benefits, fewer adverse effects, and perhaps additional positive features. As a proof-of-concept study for the inflammatory hypothesis of atherothrombosis, the CANTOS trial was a successful; however more realistic choices are needed.

## CONCLUSION

There are extensive data supporting that both high LDL-C levels and low grade arterial inflammation are implicated in the pathophysiology of atherosclerosis. Reduction of both leads to the decline in the incidence of CVD clinical manifestation. The use of statins remains the cornerstone of hypolipidaemic treatment. This is because they substantially decrease LDL-C and have pleiotropic effects (among which is a reduction in arterial wall inflammation). In some cases (*e.g.* the ARMYDA studies) the clinical benefit by statins are manifested through anti-inflammatory effects, while in some cases (PROVE IT study) with both effects. In IMPROVE IT, and other studies, significantly more patients treated with ezetimibe/simvastatin met prespecified dual LDL-C (<70 mg/dl) and hsCRP (2 mg/l) targets than patients treated with simvastatin alone [81-86]. This effect of combination therapy may result in better results than those observed with statin monotherapy [82]. Simultaneous use of statins and PCSK9i promises to reduce CVD events by lowering LDL-C levels, but a meta-analysis did not find an effect on CRP levels [87]. However, it has to be noted that many PCSK9i trials involved patients already maximally treated with statins (± ezetimibe). Therefore, a maximal lowering of CRP levels might have already occurred [88].

Canakinumab conveys its CVD event reduction only though reduction in low-grade arterial inflammation. However, it is difficult to prove that this expensive drug is cost effective given the relatively low reduction of CVD risk (15% reduction in the primary endpoint) only with the 150 mg/3 months dose and the increased risk of infections. It seems that the best choice is a potent statin with anti-inflammatory effects. The use of inexpensive generic statins is cost-effective both in primary and secondary prevention in an era where all countries have to reduce their health costs.
